# Seroprevalence of *Toxoplasma gondii* in domestic cats, dogs and rabbits from Poland

**DOI:** 10.1007/s11259-022-10055-0

**Published:** 2023-01-16

**Authors:** Hanna Turlewicz-Podbielska, Jakub Jędrzej Ruszkowski, Maciej Gogulski, Małgorzata Pomorska-Mól

**Affiliations:** 1 Department of Preclinical Sciences and Infectious Diseases, Poznan University of Life Sciences, Wolynska 35, 60‑637 Poznan, Poland; 2 Department of Animal Anatomy, Poznan University of Life Sciences, Wojska Polskiego 71C, 60‑625 Poznan, Poland

**Keywords:** Cats, Dogs, Rabbits, Seroprevalence, *Toxoplasma gondii*

## Abstract

The seroprevalence of *Toxoplasma gondii* in domestic cats, dogs and rabbits was evaluated. Samples from cats and dogs were collected from five veterinary practices from various parts of Poland - Poznan (wielkopolskie voivodeship), Przemysl (podkarpackie voivodeship), Kluczbork (opolskie voivodeship), Lublin (lubelskie voivodeship) and Deblin (lubelskie voivodeship). Moreover, the samples from rabbits were collected in Poznan. In total, serum samples from 193 cats, 204 dogs and 71 rabbits were randomly selected and tested for specific antibodies against *T. gondii* using a commercial ELISA test. Pathogen seroprevalence among cats and dogs was calculated at a 95% confidence interval (CI) for each sex and age category (up to 12 months, 1–3 years, 4–7 years and over 8 years) and compared with a chi-squared test. The highest seroprevalence of *T. gondii* was noted in cats − 49.74% (96/193; 95% CI: 42.76–56.73). In dogs, it reached 28.92% (59/204; 95% CI: 23.13–35.49). Only 1 rabbit (3-year-old male) was seropositive, and the seroprevalence in rabbits was 1.41% (1/71; 95% CI: 0.25–7.56). A statistically significant correlation between seropositivity and age (p < 0.05) was observed in cats and dogs. No statistically significant difference in seroprevalence concerning gender or location was found in cats and dogs. Our findings indicate that cat and dog serum samples had a high frequency of anti-*T. gondii* antibodies, while rabbit serum samples had low frequency and that these species are exposed to *T. gondii* in Poland and develop humoral response due to infection.

## Introduction

*Toxoplasma gondii* is a widespread protozoan and the causative agent of toxoplasmosis – a zoonotic disease. *T. gondii* represents a wide range of host organisms (Webster and Dubey 2010). Clinical manifestations of toxoplasmosis in cats are varied, thus diagnosis of feline toxoplasmosis is often challenging. Most pathologies are linked with the nervous, alimentary and respiratory systems, although the infection may be subclinical either. Dogs rarely suffer from toxoplasmosis as a primary disease. Neurological or other symptoms may occur in dogs; however, in most cases, the clinical disease is associated with immunosuppression (Calero-Bernal and Gennari 2019). *T. gondii* infection in rabbits is usually not manifested in any clinical symptoms (Woźniak-Biel et al. 2020). Dogs and cats can be involved in the maintenance of the urban and periurban life cycle of *T. gondii* and they usually share the same areas with humans. Owning a rabbit has been also recognised as a risk factor for *T. gondii* infection (Kolbekova et al. 2007). Rabbit owners may become infected when handling hay contaminated with oocysts from feline faeces. Moreover, spending time in rabbit-keeping facilities, where cats may roam freely, might also pose a threat of inhaling or ingesting oocysts (Kolbekova et al. 2007). The course of the infection in immunocompetent human individuals is usually asymptomatic or mild; however, *T. gondii* infection poses a risk for the fetus’s development of abnormalities after infection during gestation (Bieńkowski et al. 2022). Seropositivity in cats, dogs and rabbits is a result of previous exposure to this pathogen; hence these animals may serve as a potential indicator of environment contamination with *T. gondii* oocysts. The serological survey is expected to provide information that allows for the assessment of the possibility and potential frequency of the contact incidents of the host species with *T. gondii* and the evaluation of the epidemiological risk that the presence of these hosts implies for public health. The aim of the present study was to determine the seroprevalence of *T. gondii* in domestic cats, dogs and rabbits. It also examined the prevalence of specific antibodies in these species of different age groups, gender and different locations of Poland.

## Materials and methods

### Samples

**Fig. 1:**
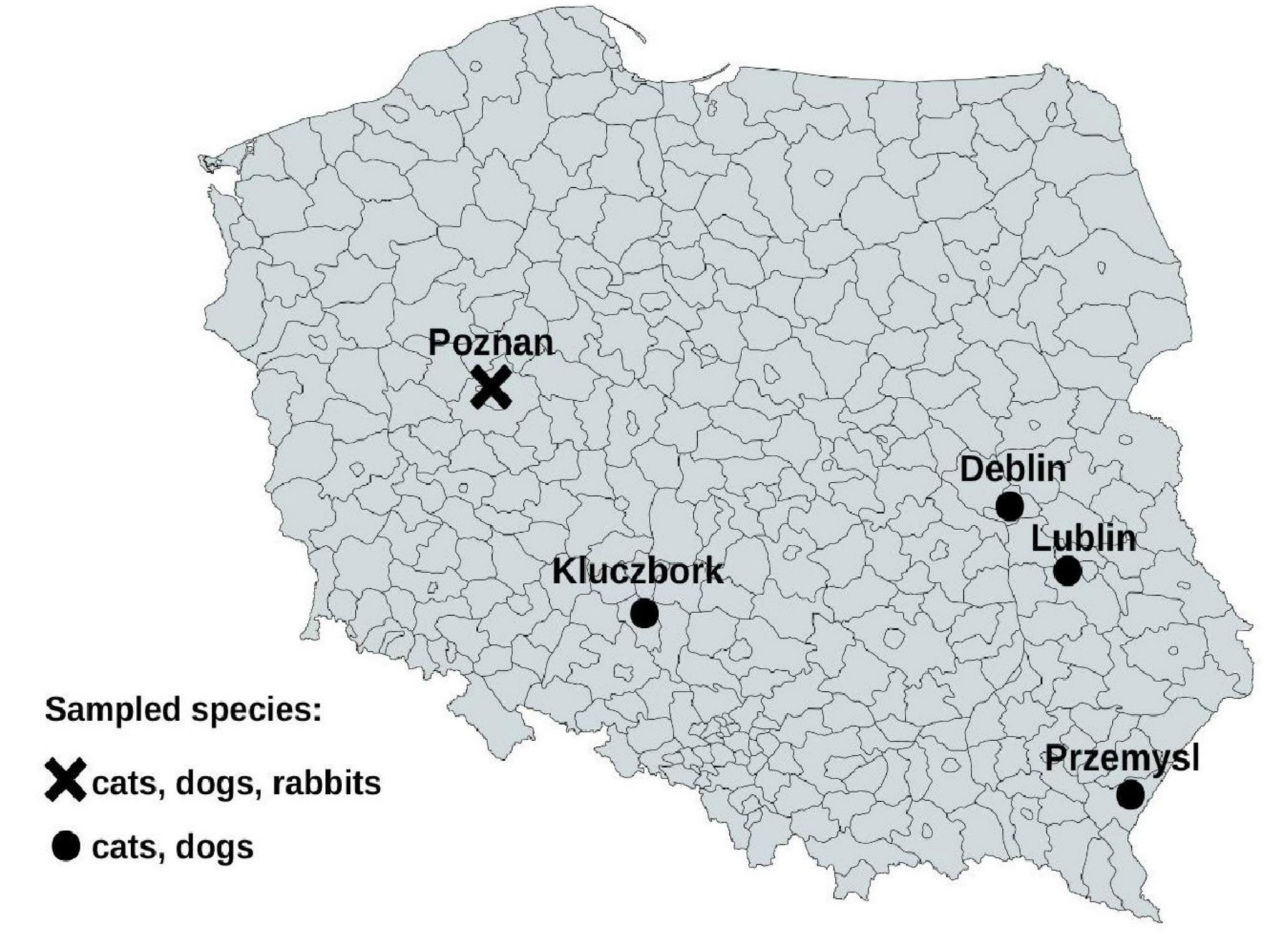
Map of sampling regions and species from which samples were collected

A total of 468 serum samples collected between September 2020 and January 2022 in five veterinary practices located in various parts of Poland (Poznan 52°24′24″N 16°55′47″E (wielkopolskie voivodeship); Przemysl 49°47′05″N 22°46′02″E (podkarpackie voivodeship); Kluczbork 18°13′E 50°58′N (opolskie voivodeship); Lublin 22°34′E 51°15′N (lubelskie voivodeship); Deblin 21°52′E 51°34′N (lubelskie voivodeship) (Fig. 1.) were selected randomly for this study. Samples from rabbits were collected in one practice (*n* = 71). Rabbit sera came from animals from Poznan and their surroundings. Sera were stored at − 70 °C until analyses. In total, serum samples from 193 cats, 204 dogs and 71 rabbits were randomly selected and used in the study. For each sample, the following information has been available: species, gender, age at sampling, location and clinical presentation (healthy, presence of gastrointestinal, respiratory or neurological disorders, other symptoms).

### Detection of *T. gondii* antibodies

An ELISA (ID Screen® Toxoplasmosis Indirect Multi-species, IDVet, Grabels, France) was used to detect anti-*Toxoplasma* antibodies according to the manufacturer`s instruction. Briefly, 10 µL of sera was diluted with 90 µL ELISA dilution buffer in the test plate pre-coated with the P30 non-infectious *T. gondii* antigen and incubated for 45 min at room temperature (21°C +/- 5°C). The plate was washed three times, and the conjugate was added to each well. Then, the plate was incubated at the same temperature for 30 min and washed three times. Next, 100 µL of 3,3’,5,5’-tetramethylbenzidine (TMB) substrate solution was added to each well for 15 min, followed by 100 µL of stop solution. The optical density (OD) was measured at 450 nm in the Infinite® 200 PRO microplate reader (TECAN, Mannedorf, Switzerland) immediately after stopping the reaction. The mean value of the measured OD value for the Positive Control (OD_PC_) and the mean value of the measured OD value for the Negative Control (OD_NC_) was used to test validation. OD_PC_ must be > 0.350 and OD_PC_/ OD_NC_ must be > 3. The ratio of the sample OD to the mean OD of the positive control (S/P) was calculated using the following equation: S/P = (OD_sample_ − OD_NC_)/(OD_PC_ − OD_NC_). Samples with S/P ≥ 0.5 were considered positive, whereas samples with S/P ≤ 0.4 were considered negative. Samples with S/P ratio > 0.4 and < 0.5 were considered as doubtful.

### Statistical analysis

The analyses were performed using RStudio (version 4.1.2), except for prevalence (with 95% confidence intervals (CI), which was available as an online program (https://epitools.ausvet.com.au/ciproportion). CI for prevalence was calculated with the Wilson score method. Pearson’s chi-square (*χ*2) tests were used to analyze the data in different species, age groups, locations and genders. The odds ratio (OR) value was calculated with a 95% CI to analyze the risk of seroconversion in different age groups in dogs and cats. A *p*-value of < 0.05 was statistically significant. The map was created using an online program (https://www.mapchart.net) and canva (https://www.canva.com).

## Results

The highest seroprevalence of *T. gondii* was reported among cats − 49.74% (96/193; 95% CI: 42.76–56.73). The seroprevalence among dogs was 28.92% (59/204; 95% CI: 23.13–35.49), while among rabbits it reached 1.41% (1/71; 95% CI: 0.25–7.56). Only 1 rabbit (a 3-year-old, clinically healthy male) was seropositive.

The correlation between the presence of anti-*T. gondii* antibodies and age observed in cats and dogs were statistically significant (*p* < 0.05). Detailed data on seroprevalence among each age group in dogs and cats and the association between age category and seroprevalence are presented in Tables 1 and 2. Our findings indicate that cats in age groups 4–7 and dogs in age group 8 + had a higher OR compared to age category < 1. Detailed information about OR in other age groups is presented in Table 2.

**Table 1:** The number of seropositive and seronegative individuals, and the prevalence of anti-T.*gondii* antibodies in different age groups in dogs and cats and the proportion of seropositive cats and dogs representing various clinical disorders

Age group (years)	Positive	Negative	Total	Seroprevalence (%)	^a^95% CI	
	Cats (n = 193)					
< 1	4	17	21	19.05	7.67-40.00	
1–3	24	24	48	50.00	36.39–63.61	
4–7	26	20	46	56.52	42.25–69.79	
8+	42	36	78	53.87	42.86–64.47	
	Dogs (n = 204)					
< 1	0	22	22	0.00	0.00-14.87	
1–3	10	25	35	28.57	16.33–45.05	
4–7	13	31	44	29.55	18.16–44.22	
8+	36	67	103	34.95	26.44–44.55	
Symptoms	(+) Total	% Positive	p-value	(+) Total	% Positive	p-value
	Cats (n = 193)			Dogs (n = 204)		
Healthy	(22) 49	44.90	0.77	(15) 55	27.27	0.44
Gastrointestinal disorders	(9) 21	42.86		(10) 35	28.57	
Respiratory disorders	(8) 14	57.14		(4) 11	36.36	
Neurological disorders	(6) 12	50.00		(5) 9	55.56	
Other	(51) 97	52.58		(25) 94	26.60	

^a^95% CI: lower and upper values for the 95% confidence interval.

**Table 2:** Odds ratio in different age categories of dogs and cats

Age group (years)	OR^b^	^a^95% CI	*p*-value
Cats (n = 193)			
< 1	Ref^c^		
1–3	4.24	1.25–14.50	< 0.05
4–7	5.53	1.61–19.01	< 0.01
8+	4.96	1.43–16.08	< 0.01
Dogs (n = 204)			
< 1	Ref^c^		
1–3	18.52	1.03-334.47	< 0.05
4–7	19.29	1.09-341.54	< 0.05
8+	24.33	1.43-412.86	< 0.05

^a^95% CI: lower and upper values for the 95% confidence interval

^b^OR: odds ratio

^c^Ref: reference category

Gender difference in seroprevalence was found between feline males (51.52%; 51/99; CI: 41.80-61.12) and females (47.87%; 45/94; CI: 38.06–57.85), however, it was not statistically significant (*p* > 0.05). In dogs, the gender difference in seroprevalence was not statistically significant either (*p* > 0.05). The seroprevalence among males was 31.00% (31/100; CI: 22.78–40.63) and in females – 26.92% (28/104; CI: 19.33–36.16). The association between clinical picture and seropositivity is presented in Table 1, although it was not statistically significant.

Differences in seroprevalence in relation to location were not statistically significant in cats (*p* > 0.05) and dogs (*p* > 0.05). Detailed data about samples that tested positive for anti-*T. gondii* antibodies in relation to samples obtained from cats, dogs and rabbits in each location in Poland are presented in Table 3. The S/P values in cats considered seropositive ranged from 0.51 to 2.29 (mean 1.39.50 ± 16.58). The S/P values in dogs considered as seropositive ranged from 0.51 to 2.77 (1.35 ± 0.58). The S/P value in seropositive rabbit was 3.11.

**Table 3:** Seroprevalence of anti-*T. gondii* antibodies in cats, dogs and rabbits in relation to samples obtained from each species in each location of Poland

Location	Positive	Negative	Total	Seroprevalence (%)	95% CI^a^
Cats (n = 193)					
Deblin	5	7	12	41.67	19.33–68.05
Kluczbork	4	4	8	50.00	21.52–78.48
Lublin	32	30	62	51.61	39.45–63.69
Poznan	48	47	95	50.53	40.65–60.36
Przemysl	7	9	16	43.75	23.10-66.82
Dogs (n = 204)					
Deblin	5	21	26	19.23	8.51–37.88
Kluczbork	8	14	22	36.36	19.73–57.05
Lublin	16	31	47	34.04	22.17–48.33
Poznan	26	68	94	27.66	19.63–37.44
Przemysl	4	11	15	26.67	10.90-51.95
Rabbits (n = 71)					
Poznan	1	70	71	1.41	0.25–7.56

^a^95% CI: lower and upper values for the 95% confidence interval

## Discussion

Cats are the sole definitive hosts of *T. gondii* and excrete the oocysts with faeces, which may spread over and lead to surface and groundwater contamination. The seroprevalence among cats obtained in the present study was lower than in the study performed earlier in other location of Poland: Olsztyn city (Michalski et al. 2010), surroundings of Lublin city (Sroka et al. 2010) and silesian voivodeship (southwestern Poland) (Sroka et al. 2018). The authors examined domestic cats in an urban area in Olsztyn, Poland and 68.1% (92/135) of the tested animals were seropositive (Michalski et al. 2010). The seroprevalence among cats from the surroundings of Lublin city reached 75% (33/44) (Sroka et al. 2010) and 68.8% (143/208) in silesian voivodeship. To date, the seropositivity decreased, and probably fewer animals came into contact with the pathogen over time. Moreover, cats included in the previous studies inhabited the surroundings and rural areas of Lublin city and rural areas of silesian voivodeship, where the exposure to *T. gondii* could be higher than in our study. Our results are consistent with those obtained in other European countries: Norway and Finland, where the seroprevalence rates in cats were 41.0% (196/478) and 48.4% (237/490) respectively (Jokelainen et al. 2012; Sævik et al. 2015) and higher to those obtained in Belgium and Spain where it was 24.9% (171/567) and 32.3% (189/585), respectively (De Craeye et al. 2008; Miro et al. 2004).

Dogs may become infected with *T. gondii* as intermediate hosts by ingesting tissue cysts in raw meat or sporulated oocysts in the contaminated ground (Dubey, 2010). Our study results were consistent with those conducted in Austria, where 26% (63/242) of the investigated dogs were seropositive (Wanha et al. 2005). In Portugal, the overall seroprevalence in dogs was higher and reached 38% (256/673) (Lopes et al. 2011). In our study, both in dogs and cats, differences observed in seropositivity in relation to different locations in Poland were not statistically significant. All samples came from domestic animals from the cities and not from rural areas, which could be a reason for the lack of variance in seropositivity. Our results showed that more than 1/4 of the examined dogs came into contact with *T. gondii*. The differences in the prevalence of anti-*T. gondii* antibodies in each Europe country may be due to ecological and geographical factors, the number of stray animals, feeding habits and animal welfare conditions for animals in these areas.

Rabbits are mainly infected with *T. gondii* via the ingestion of contaminated water and plants containing oocysts excreted by domestic cats or free-ranging felids with which they share the same habitats (Dubey, 2010). However, the seropositive rabbit in our study had no direct contact with felids or other animals and was kept indoors. Feeding with unwashed or inadequately washed plants contaminated with oocysts may be a potential cause of infection with *T. gondii*. In our study, the seroprevalence in rabbits was lower than in a previous study conducted in Poland, where antibodies against *T. gondii* were found in 12.12% (44/360) of pet rabbit samples (Woźniak-Biel et al. 2020). Their results are more consistent with the studies including farm and wild rabbits, which are expected to be more exposed to *T. gondii* infection. In farm rabbit samples collected throughout the Czech and Slovak Republic, the seroprevalence of *T. gondii* in 6 commercial farms was 0.4% (4/902), and 10.1% (99/981) in the 29 household farms (Neumayerová et al. 2014). The low seroprevalence among rabbits in our study is probably due to the fact that all rabbit sera came from rabbits kept indoors.

The correlation between increasing age and the presence of anti*-T. gondii* antibodies was statistically significant in several studies concerning cats, dogs and rabbits (Arruda et al. 2021; Lopes et al. 2011; Sævik et al. 2015; Wang et al. 2018; Sroka et al. 2018). In our study, the OR varied from 6.67 to 7.46 and was the highest in age group 3–7, indicating that age is a risk factor for seropositivity among dogs and cats. Higher seroprevalence of anti-*T*.*gondii* antibodies was also noted in older rabbits compared to younger ones (Wang et al. 2018). In contrast, Woźniak-Biel et al. did not observe statistical differences in seroprevalence according to the age of the rabbits (Woźniak-Biel et al. 2020). Only one seropositive rabbit was detected in our study; hence analysis of differences in seroprevalence according to age was not possible.

Results of our study indicate that the seroprevalence of *T. gondii* in dogs and cats in different locations of Poland is high (higher in cats), and the percentage of seroreagents increases with age. Additionally, dogs, cats and rabbits are exposed to the pathogen, can become infected and develop a humoral response as a result of infection with *T. gondii*. These results are beneficial to researchers, health workers and veterinarians. Control measures are required both to reduce the exposure of felids and intermediate hosts to infective oocysts and reduce the shedding of oocysts into the environment by definitive hosts. Careful handling and disposal of feline faeces and the use of disinfectant are suggested in living areas if they become contaminated with feline faeces. Moreover, prevention of infection in cats may reduce environmental pollution from *T. gondii* oocysts, which will have a positive impact on intermediate host animals and public health. This can be achieved by keeping cats indoors and feeding cats commercially prepared cooked foods. Cats should not be allowed to eat uncooked meat or hunt and eat intermediate hosts, such as rodents.

## Data Availability

The data that support the findings of this study are not openly available but are available from the corresponding author upon reasonable request.
